# Effect of gap junctions on RAW264.7 macrophages infected with H37Rv

**DOI:** 10.1097/MD.0000000000012125

**Published:** 2018-08-21

**Authors:** Yang Lu, Xin-min Wang, Pu Yang, Ling Han, Ying-zi Wang, Zhi-hong Zheng, Fang Wu, Wan-jiang Zhang, Le Zhang

**Affiliations:** a Department of Pathophysiology/the Key Laboratories for Xinjiang Endemic and Ethnic Diseases; b Department of Urinary Surgery, The First Affiliated Hospital, Medical College of Shihezi University, Shihezi, Xinjiang, China.

**Keywords:** connexion, GJIC, H37Rv infection, immune defense response, macrophage

## Abstract

**Background:**

Apoptosis and inflammation have been shown to play an important role in the mechanisms involved in the pathogenesis of *Mycobacterium tuberculosis* (MTB) infection. When macrophages undergo apoptosis and polarization, gap junctions (GJs) may be needed to provide conditions for their functions. Connexin 43 (Cx43) and connexin 37 (Cx37) are the main connexins in macrophages that participate in the formation of GJ channels.

**Methods:**

An H37Rv infection RAW264.7 macrophage model was established to investigate the associate between connexins and host macrophage immune defense response after MTB infection. First, Real-time Polymerase Chian Reaction (RT-PCR) was used to detect the mRNA expression of Cx43 and Cx37. Cx43 protein expression and location was detected by western blotting and immunofluorescence. Confocal microscope was used to assay the gap junctional intercellular communication (GJIC). Then, electron microscope used to observe the morphology of macrophages. Finally, RAW264.7 macrophage apoptosis and mitochondrial membrane potential was detected by flow cytometry, and the expression of inflammation factors such as CD86, CD206, and IL-6, IL-10, TNF-α, and TGF-β were detected by Real-time PCR and enzyme-linked-immunosorbent serologic assay (ELISA).

**Results:**

H37Rv infection significantly promoted host macrophage Cx43 mRNA and protein expression (increased 1.6-fold and 0.3-fold respectively), and enhanced host macrophage GJIC. When host macrophage cell-to-cell communication induced by H37Rv infection, the apoptosis rate and inflammatory factors expression also increased.

**Conclusions:**

The results confirm that H37Rv infection can obviously induce host macrophage Cx43 expression and enhance GJIC, which may implicated in host macrophage inflammatory reaction, to regulate the release of inflammatory factors and/or initiate apoptosis to activate host immune defense response.

## 1 Introduction

Tuberculosis (TB) is one of the world's most harmful infectious diseases, and human immunodeficiency virus (HIV) co-infection has made it more complicated. The manner by which the macrophage responds following infection by *Mycobacterium tuberculosis* (MTB) makes a crucial contribution to the host immune response and the outcome of infection. Evidence suggests that macrophages present the first line of defense against MTB.^[1]^ When MTB encounters a host, the host macrophages trigger apoptosis and release inflammatory factors to eliminate MTB pathogens, which are the important host immune defense mechanisms.^[2,3]^ Thus, increasing reports have explored the mechanisms between MTB and host macrophage, but the cell-mediated immune responses in host macrophages are still unclear.

Gap junctions (GJ) and connexins are present in the immune system. It is the only membrane channel that can allow the direct communication between neighboring cells. It has been reported that connexin 37 (Cx37) and connexin 43 (Cx43) are widely distributed GJ proteins in macrophages.^[4]^ Macrophage clusters are always in tissues affected by different inflammatory disease states, in which cell-to-cell proximity may allow the intercellular contacts necessary to accomplish some relevant functions.^[5]^ Eliseo et al^[5]^ reported that the transient expression of Cx43 by monocyte/macrophage cells enables the formation of GJ channels that allow the intercellular conversation required for different cellular responses and functions during inflammation.

Although connexin-based channels have been identified in many immune cells,^[6,7]^ their role in RAW264.7 macrophages in the regulation of the MTB-infection inflammatory response remains unclear. Thus, we aimed to analyse the effects of cell-to-cell communication and immune response during H37Rv infection.

## 2 Materials and methods

### 2.1 Cells

RAW264.7 cells were purchased from the Shanghai Institute of Biochemistry and Cell Biology (Shanghai, China). The cells were cultured in DMEM (HyClone) medium with 10% FBS (HyClone) and 1% penicillin/streptomycin and incubated at 37°C in 5% CO_2_.

### 2.2 Bacteria

H37Rv strains were purchased from Chinese Medicine Biological Products (Beijing, China). The bacteria were mixed with Sutong culture and normal saline (0.5 mL; volume ratio of 3:1), and 100 μL bacterial dilutions were spread on modified Roche medium. After 2 to 3 weeks, the bacterial strains that grew well on the Roche medium slant were ground and a small amount of saline solution containing 0.05% Tween-80 was added. The bacterial suspension was adjusted to the McFarland standard corresponding to 1 × 10^7^ colony-forming units (CFU)/mL.

### 2.3 H37Rv infection RAW264.7 cell model construction

Uninfected RAW264.7 cells were used as a control. Groups of RAW264.7 cells were infected with H37Rv strains at a multiplicity of infection (MOI) of 10:1 (bacterial:cell) and incubated at 37°C in a 5% CO_2_ incubator. The infected cells were washed with PBS 3 times in 4 hours (time 0) after H37Rv infection, then added to fresh medium with 10% FBS.

### 2.4 Real-time PCR

Total RNA was isolated from the RAW264.7 cells using RNeasy kit (TIANGEN, Beijing, China) following the manufacturer's protocol. First-strand cDNA was synthesized using a Thermoscript RT kit (Life Technologies, Rockville, MD). Primers were synthesized by Sangon Biotech (Shanghai, China). Samples were amplified with a QuantiFast SYBR Green PCR kit (QIAGEN, Hilden, Germany) according to the manufacturer's instructions. Relative amounts of transcripts were calculated using the 2^−ΔΔCt^ formula. The primers used for RT-PCR were as follows: Cx37: forward 5′-GCCAACTTGACCACAGAGGA-3′, reverse 5′-CTTGGATGCAGAGCTGTTGG-3′; Cx43: forward 5′-AGAGGTGGCCTGCTGAGAAC-3′, reverse 5′-GCAGGTGTAGACCGCACTCA-3′; iNOS: forward 5′-TGATGTGCTGCCTCTGGTCT-3′, reverse 5′-GAGCTCCTGGAACCACTCGT-3′; CD86: forward 5′-ACTGGACTCTACGACTTCACAATGTTC-3′, reverse 5′-AAGTTGGCGATCACTGACAGTTCTG-3′; CD206: forward 5′-GTCTGAGTGTACGCAGTGGTTGG-3′, reverse 5′-TCTGATGATGGACTTCCTGGTAGCC-3′; β-actin (as control): forward 5′-GTGACGTTGACATCCGTAAAGA-3′, reverse 5′-GCCGGACTCATCGTACTCC-3′.

### 2.5 Western blotting

After infection with H37Rv strains for 12 hours, the control and infected RAW264.7 cells were collected and lysed using RIPA lysis buffer (Solarbio Biotechnology, Beijing, China). RAW264.7 cell homogenates containing equal amounts of protein were separated using 10% SDS-polyacrylamide gel electrophoresis (SDS-PAGE) and assayed by western blotting as described previously.^[8]^ A concentration of rabbit anti-mouse Cx43 primary antibodies (1:1000) was purchased from Cell Signaling Technology (Danvers, MA), and mouse anti-mouse β-actin (1:1000) was purchased from ZSGB-BIO (Beijing, China). The secondary antibodies (1:20000, horseradish peroxidase-conjugated goat anti-rabbit or goat anti-mouse secondary antibodies) were purchased from ZSGB-BIO (Beijing, China). Immunoblots were developed using an enhanced chemiluminescent reaction (ECL, Millipore, Burlington, MA).

### 2.6 Immunofluorescence

For the immunofluorescence assay, both the control and infected cells were seeded on cover slips and incubated at 37°C until 40% to 50% confluence. They were then fixed and permeabilized using an Intracellular Fixation and Permeabilization Buffer kit (eBioscience, San Diego, CA) for 20 minutes at room temperature. The cells were blocked in 10% (w/v) normal goat serum in PBS for 30 minutes at room temperature. Next, the cells were incubated for 2 hours at 37°C with Cx43 antibodies (Cell Signaling Technology), followed by goat anti-rabbit FITC-conjugated IgG, and Rhodamine was used to label the cell nuclei (ZSGB-BIO, China). Digital pictures were taken using a Zeiss LSM 510 Meta Laser scanning confocal microscope.

### 2.7 Gap junctional intercellular communication assay

The cell-to-cell GJ communication assay protocol was performed as described previously.^[9,10]^ The donor and recipient cells were differentially labeled for 30 minutes with the GJ-permeable dye, calcein AM, and lipophilic dye DiIC 18 (Molecular Probes, Eugene, OR) in DMEM containing 10% FBS, respectively. The donor cells were trypsinized after being washed and added to acceptor cells at a 1:5 (donor: acceptor) ratio for 3 hours at 37°C in 5% CO_2_. Co-cultures were harvested and subjected to a confocal microscope (ZEISS, LSM 510). If gap junctional intercellular communication (GJIC) occurred, calcein was transferred from the donor cells (green) to the recipient cells (red), and the double-labeled cells represented the communicating cells.^[10]^

### 2.8 Scanning electron microscope

The RAW264.7 cells were collected and washed with PBS 3 times, then fixed in 2.5% glutaraldehyde solution, dehydrated through a graded tert-butanol series to 100%. The samples were coated with platinum sputtering after retaining it under a vacuum condition and imaged using a scanning electron microscope (SEM) (JEOL, JSM-6390LV).

### 2.9 Transmission electron microscopy

The RAW264.7 cells were collected and washed with PBS 3 times then fixed in 2.5% glutaraldehyde solution and osmium tetroxide, dehydrated through a graded ethanol series to 100%, and polymerized in epoxy resin. Ultrathin sections were collected, stained, and imaged using a transmission electron microscope (JEOL, transmission electron microscopy [TEM]-1230).

### 2.10 Annexin V-APC/7-AAD cytometry analysis

The control and infected RAW264.7 cells were collected and washed 3 times with cold PBS. The methods of dyeing were performed according to the instructions until they were completely stained. The cells were resuspended in cold 1× binding buffer to a concentration of 1 × 10^6^, 100 μL of cells were added to each tube, and then 5 μL of Annexin V-APC and 10 μL of 7-AAD were added. The mixture was mixed gently and incubated for 15 minutes on ice. The cells were not washed and 380 μL of cold 1× binding buffer were added to each tube for analysis using a flow cytometer (BD FACSCanto II).

### 2.11 Mitochondrial membrane potential assay

The mitochondrial membrane potential (ΔΨm) was determined using a JC-10 kit according to the manufacturer's manual (KeyGEN BioTECH, Jiangsu, China). A total of 1 × 10^6^ macrophages were stained with JC-10 working fluid at 37°C in a 5% CO_2_ incubator for 20 minutes. Polarized mitochondria with J-aggregate forms and depolarized mitochondria with monomer forms were marked by red and green fluorescence staining, respectively. They were analyzed using flow cytometry (BD FACSCanto II) with appropriate gate settings in the FL-1 (green) and FL-2 (red) channels.

### 2.12 Enzyme-linked-immunosorbent serologic assay

The supernatant was collected 12 hours after H37Rv infection. IL-6, IL-10, TNF-α, and TGF-β were measured using enzyme-linked-immunosorbent serologic assay (ELISA) kits (Elabscience, Wuhan, China) according to the manufacturer's instructions.

### 2.13 Institutional review board approval

This study was an in vitro experiment. The Internal Review Board of the Shihezi University reviewed and approved the study protocol.

### 2.14 Statistical analysis

The data are expressed as mean± SD from 3 independent experiments. Statistical correlation of the data was checked for significance using Student's *t* test. Differences were considered statistically significant at *P* < .05. The analyses were performed using SPSS 20.0 software.

## 3 Result

### 3.1 H37Rv infection changes connexins in RAW264.7 macrophages

**Figure 1:**
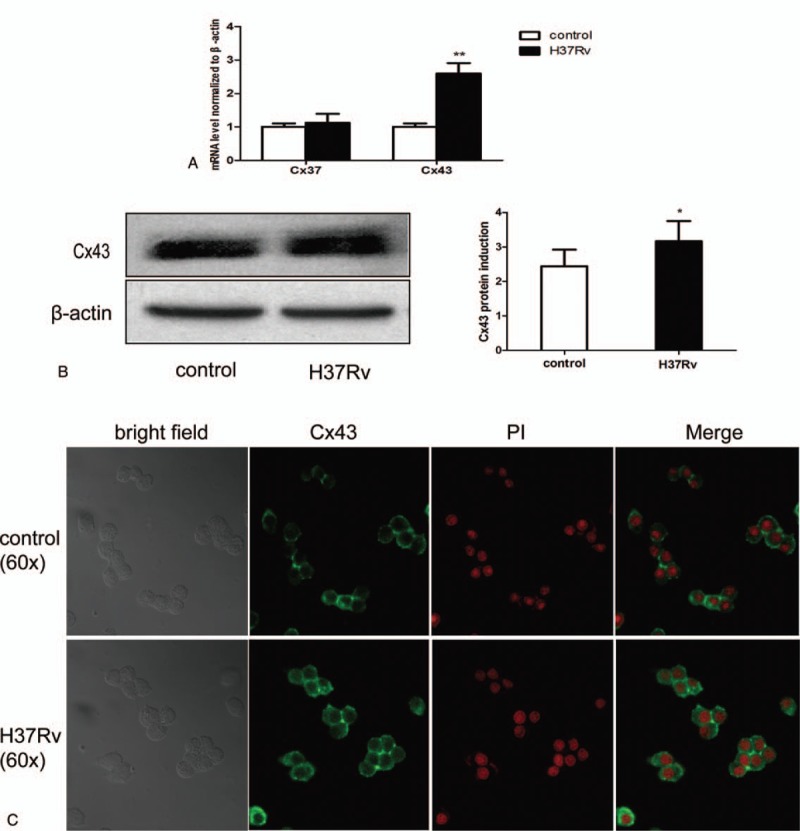
H37Rv infection induces host macrophage connexin expression. RAW264.7 cells were infected by H37Rv strains for 12 hours. Total RNA and protein were tested using RT-PCR (Cx37 and Cx43 mRNA) (A) and Western blotting (Cx43 protein) (B). Both the control and infection cells were collected and tested using immunofluorescence analysis (C). Data are expressed as mean±SD from 3 independent experiments. ^∗^*P* < .05 versus control; ^∗∗^*P* < .01 versus control.

The mRNA of Cx37 and Cx43 from the control and the H37Rv-infected RAW264.7 macrophages was analyzed using RT-PCR to determine whether the connexins contribute to the inflammatory pathogenesis of MTB infection. Cx43 protein and immunofluorescence were also investigated. As shown in Figure 1, after infection by H37Rv strains, Cx37 and Cx43 mRNA increased compared to the control. Cx43 mRNA increased 1.6-fold, and Cx43 protein increased 0.3-fold. From the immunofluorescence results, Cx43 in the normal group of RAW264.7 cells was mainly distributed in the cell membrane. In the infected cells, the green fluorescence of the cell membrane was significantly enhanced, indicating that more Cx43 appeared (Fig. 1).

### 3.2 H37Rv infection induces host macrophage cell-to-cell GJ communication

**Figure 2:**
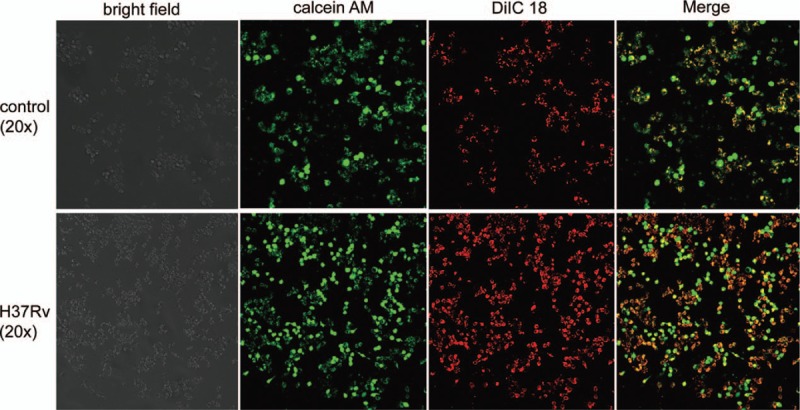
H37Rv infection enhances host macrophage GJIC. After 12 hours, both the control and the H37Rv-infected RAW264.7 cells were collected and stained with calcein AM and DiIC 18 and then subjected to a confocal microscope for GJIC assay. GJIC = gap junctional intercellular communication.

It is well known that Cx43 exerts its function through GJ-mediated pathways, and our results showed that H37Rv infection significantly induced host macrophage Cx43 expression. Therefore, we investigated whether H37Rv infection promotes the exchange of GJ communication in host macrophages. GJ formation in the infection group dramatically increased. These results suggest that H37Rv infection can promote host macrophage cell-to-cell communication through GJ (Fig. 2).

### 3.3 H37Rv infection induces host macrophage apoptosis

**Figure 3:**
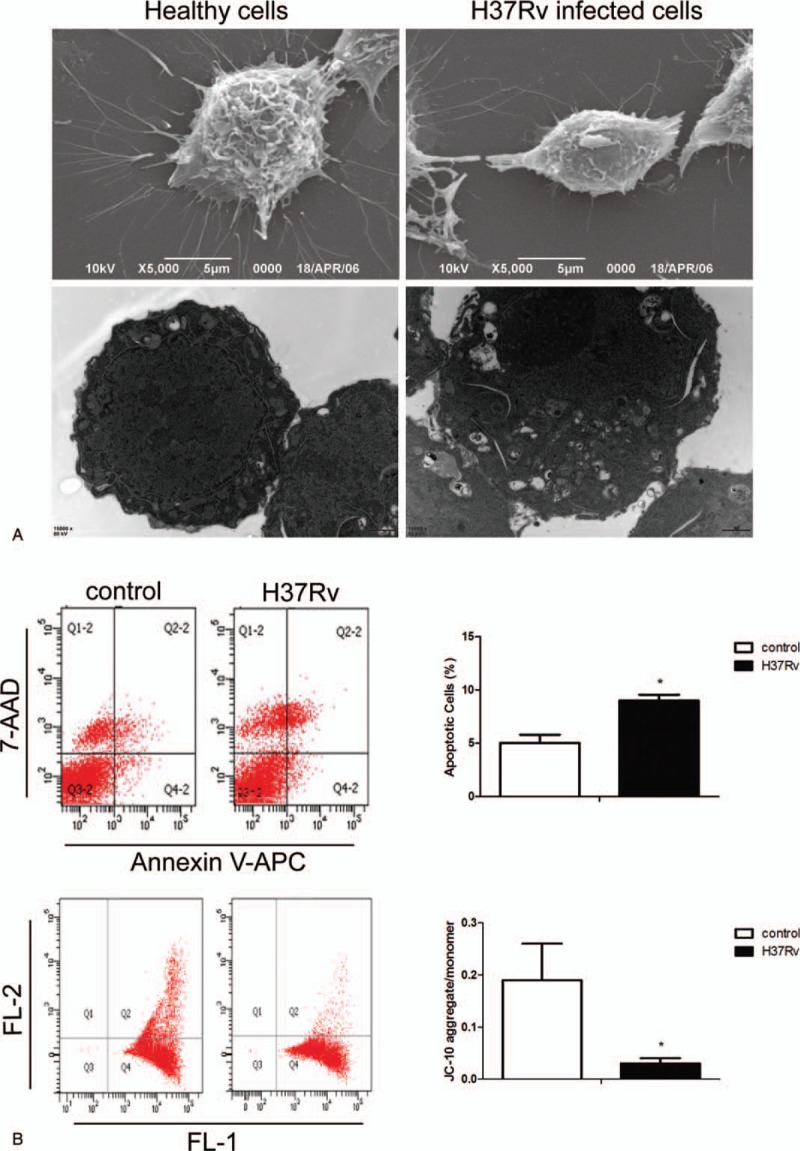
H37Rv infection promotes RAW264.7 cell apoptosis. Control and H37Rv-infected RAW264.7 macrophages were collected, and SEM and TEM were used to detect cell morphology (A). The apoptosis rate and mitochondrial membrane potential were analysed using flow cytometry (B). Data are expressed as mean±SD from 3 independent experiments. ^∗^*P* < .05 versus control. SEM = scanning electron microscope, TEM = transmission electron microscopy.

We next investigated the cell death of host macrophages following H37Rv infection. The apoptotic morphology assay of host macrophages was confirmed by morphological analysis using SEM and TEM. Healthy cells have a strong cell membrane and abundant surrounding microvilli and uniform cytoplasm, as well as a clear nuclei membrane boundary. When cells were exposed to H37Rv strains, there was a loss of microvilli, a protrusion of the plasma membrane (blebs) with apoptotic bodies, and autophagic and phagocytic vacuoles were induced in the host macrophages. Flow cytometry analysis further revealed an increased frequency of apoptosis (79%) in the infection group compared to the control. Furthermore, the JC-10 aggregate/monomer ratio was used to present the degree of depolarization of the mitochondrial membrane potential. The results also showed significant loss of mitochondrial membrane potential in the infection group (Fig. 3).

### 3.4 Increased expression of connexins and functionality of GJ in H37Rv-infected RAW264.7 cells correlates with inflammatory factor release

**Figure 4:**
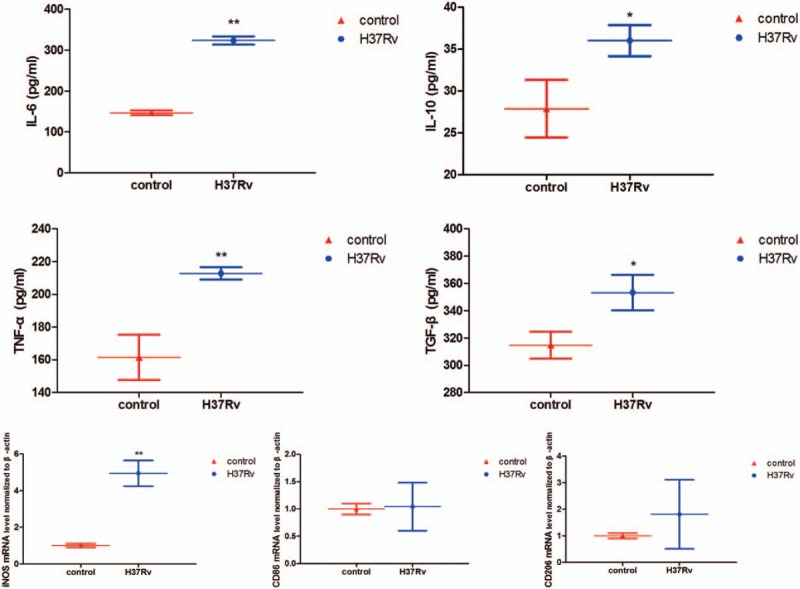
Inflammatory response triggered by H37Rv infection. RAW264.7 macrophages were infected with H37Rv for 12 hours, and then cell culture supernatant and total RNA were collected to detect IL-6, IL-10, TNF-α, TGF-β, iNOS, CD86, and CD206 levels. Data are expressed as mean ± SD from 3 independent experiments. ^∗^*P* < .05 versus control; ^∗∗^*P* < .01 versus control.

To further examine whether the GJ-dependent mechanism was involved in the inflammation response after H37Rv infection, inflammation-related factors were tested using ELISA and RT-RCR. The results indicated that IL-6, IL-10, TNF-α, TGF-β, and iNOS expression in the infection group was significantly higher than in the control group, an increase of 1.2-fold, 0.29-fold, 0.32-fold, 0.12-fold, and 3.95-fold, respectively, as well as an increase of CD86 (*P* = .891) and CD206 (*P* = .303). Thus, we speculate that the release of inflammatory factors may correlate with connexin-based GJ formation (Table 1 and Fig. 4).

**Table 1:**
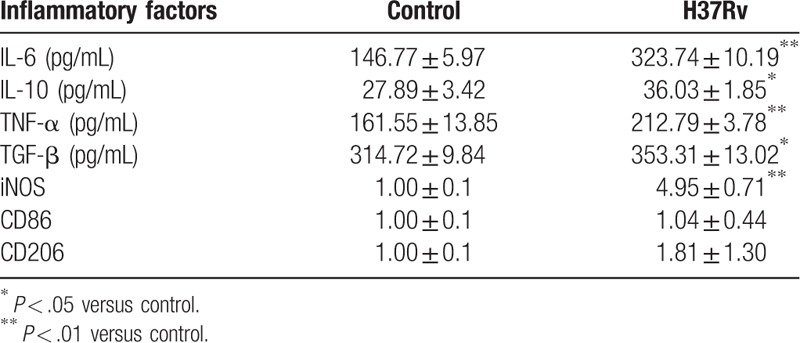
Inflammatory factor levels.

## 4 Discussion

Studies^[6,7,11]^ have confirmed the regulatory role of connexin-based cell-to-cell communication in many inflammatory diseases, but there is still no related research on MTB infection. Therefore, the current study aimed to elucidate the links between GJIC and the host macrophage immune response in H37Rv infection.

TB has become a global disease burden because the BCG vaccine provides poor immune protection and there is an increasing incidence of drug-resistant MTB.^[12,13]^ What's more, HIV has been proposed to exacerbate MTB pathogenesis via multiple mechanisms,^[14]^ and study also demonstrated that intracellular MTB could active replication of HIV,^[15]^ which made it more complicated for TB therapeutic. So fully understanding the mechanism of TB and finding new ways to treat it is urgent, as well as MTB/HIV co-infection. GJs are intercellular channels that are formed by the connexin families to allow electrical and chemical communication.^[16,17]^ The calcein transfer from each pre-loaded cell can certify the existence of GJIC through pre-labeled isolated cells by 2 fluorescent dyes: calcein, which is able to pass through the gap-junction channels, and DiIC, which is unable to pass.^[18]^ The results of the current study show that H37Rv infection can induce Cx37 and Cx43 expression (especially Cx43). Moreover, an important implication of our data is obviously enhanced GJIC (the dye transfer was rendered fluorescent yellow) in H37Rv-infected RAW264.7 macrophages, more than in the control cells. These results allow us to speculate that Cx37, Cx43, and/or other connexins compose the GJs channels and permit the direct exchange of small molecules between host macrophages, further regulating the host immune defense response. Furthermore, work from Eugenin et al found that pro-apoptotic signals that spread to neighboring uninfected cells via Cx43 containing channels causing, which “bystander apoptosis” despite absence of viral replication in HIV infection.^[19–21]^ Based on these findings, we speculate that Cx43 and GJs channels may act as a new target in deeply understanding the mechanism of MTB/HIV co-infection.

Studies have shown that macrophages are phagocytes at the frontline of the host immune defense against MTB pathogens,^[1]^ host macrophage apoptosis, and release of inflammatory cytokines as an important defense response to resist MTB pathogens.^[3,22,23]^ In many cell types, GJs can transmit apoptotic signals to control the destiny of cells that are associated with the expression of Cx43.^[24–26]^ Furthermore, researchers^[10]^ have reported that Cx43 induces apoptosis of pancreatic cancer cells and apoptosis through the mitochondrial apoptotic pathway. Our previous study showed that the different virulence of MTB infection could induce host macrophage apoptosis in vivo^[27]^ and that apoptosis may be due to the activation of the mitochondrial apoptotic pathway.^[28]^ In this in vitro study, H37Rv infection induced host RAW264.7 macrophage Cx43 expression, and we also found an increasing apoptosis ratio and mitochondrial dysfunction. Based on these results, we presume that the apoptosis of host macrophages induced by H37Rv infection is regulated by Cx43, and Cx43 exerts its function through GJ-mediated pathways. Losa et al^[29]^ found that Cx43 induction by CF-associated pathogen *Pseudomonas aeruginosa* infection epithelial cells can be considered a component of the response of the airway epithelial cells to innate immune activation, leading to a regulated increase in GJIC. Cx43 contributes to airway epithelial cell apoptosis but does not regulate IL-8 secretion; in the connection between cell-to-cell communication and the airway response, the tightly regulated expression of Cx43 may confer in normal airway epithelia a mechanism to balance the inflammatory and apoptotic responses. Studies^[7,30]^ have also shown that the functionality of GJIC was regulated by inflammatory stimulators, and inflammatory factors could upregulate the expression of connexin on the surface and enhance GJIC in cells of the immune system.^[31]^ In contrast to previous studies, our results showed that H37Rv infection led to a higher level of inflammatory cytokines such as IL-6, IL-10, TNF-α, TGF-β, and iNOS compared to the control group. Therefore, H37Rv may induce the formation of connexin channels in host macrophages and enhance GJIC, which may permit the release of inflammatory cytokines.

Taken together, the present results illustrate the importance of GJIC in MTB infection that mediates the host macrophage immune response. We speculate that Cx43 may be involved in the regulation of the production of inflammatory cytokines and host macrophage apoptosis through GJIC during H37Rv infection; the specific mechanism needs further study. A deeper understanding of GJIC molecular mechanisms that regulate MTB infection is crucial to establish a cell-based delivery system as a new approach for the therapeutic treatment of TB. Our work also provides new insights into MTB/HIV co-infection; further work still needed to describe the mechanisms.

## Author contributions

Conceived and designed the experiments: Yang Lu, Le Zhang.

Performed the experiments: Yang Lu, Pu Yang, Ling Han.

Funding acquisition: Le Zhang.

Contributed reagents/ materials/ analysis tools: Zhi-hong Zheng, Fang Wu, Wan-jiang, Zhang

Analyzed the data: Yang Lu, Ying-zi Wang.

Writing - original draft: Yang Lu, Xin-min Wang.

Writing - review & editing: Yang Lu, Xin-min Wang, Le Zhang.

**Conceptualization:** Xin-min Wang.

**Data curation:** Yang Lu.

**Funding acquisition:** Le Zhang.

**Investigation:** Ling Han.

**Methodology:** Yang Lu, Ying-zi Wang, Zhi-hong Zheng.

**Project administration:** Le Zhang.

**Resources:** Xin-min Wang, Fang Wu, Wan-jiang Zhang, Le Zhang.

**Software:** Yang Lu.

**Supervision:** Pu Yang, Ling Han.

**Writing – original draft:** Yang Lu.
